# The Relationship between Plant-Based Diet Indices and Sleep Health in Older Adults: The Mediating Role of Depressive Symptoms and Anxiety

**DOI:** 10.3390/nu16193386

**Published:** 2024-10-05

**Authors:** Junping Liu, Zhaoyue Liu, Yue Zhou, Lin Wu, Nan Wang, Xinru Liu, Yaping Liu, Xinle Yin, Aiying Yang, Libo Liang

**Affiliations:** 1 Department of Social Medicine, School of Health Management, Harbin Medical University, No. 157 Baojian Road, Nangang District, Harbin 150081, China; 2 Department of Cell Biology, School of Basic Medical Sciences, Harbin Medical University, No. 157 Baojian Road, Nangang District, Harbin 150081, China

**Keywords:** plant-based dietary pattern, sleep health, older adults, anxiety, depression

## Abstract

Background: The goal of our research was to determine the effects of plant-based dietary patterns on sleep health among older adults and to examine the parallel mediated effects of anxiety and depression. Methods: This investigation utilized data obtained from the 2018 Chinese Longitudinal Healthy Longevity Survey (CLHLS) and contained 6853 participants. Logistic regression and the restricted cubic splines (RCSs) model were employed to examine how plant-based dietary patterns affect sleep health. Additionally, Amos 26.0 was used to construct a structural equation model to examine the parallel mediated effects of anxiety and depression. Results: A higher plant-based diet index (PDI) was connected to higher odds of better sleep quality (OR = 1.209, 95% CI: 1.039–1.407) and sleep duration (OR = 1.241, 95% CI: 1.072–1.437). Conversely, an elevated unhealthy plant-based diet index (uPDI) was correlated with a lower likelihood of sleep quality (OR = 0.678, 95% CI: 0.574–0.800) and sleep duration (OR = 0.762, 95% CI: 0.647–0.896). The RCSs regression further identified a significant dose–response relationship. Mediation analysis confirmed that anxiety and depression partially mediate the relationship between plant-based diets and sleep health. Conclusions: Our study exhibited significant correlations between plant-based diets and sleep health in the elderly. Depression and anxiety were determined as parallel mediators between plant-based diets and sleep health. Controlling early dietary patterns and affective disorder could help improve sleep quality in older adults.

## 1. Background

Sleep disorders remain a public health concern, with epidemics occurring in different countries and regions [1,2]. A significant portion of the global population is troubled by sleep-related issues, such as insomnia or difficulty staying asleep. Research evidence has also highlighted the global uptrend in sleep problems, affecting both the broader public and healthcare environments [3]. Poor sleep health may lead to impairments in neurological and psychological systems, like depression and cognitive decline, as well as fatigue, which may result in a diminished life quality [4,5,6]. As the elderly population expands, health issues among older adults are gaining increasing public attention [7]. Older adults often face a variety of issues, including physical ageing, chronic diseases, sleep disorders, and psychological problems [8]. Epidemiological evidence had identified that older adults are more susceptible to poor sleep health and face greater challenges in maintaining healthy sleep patterns [9]. Sleep health among older adults is influenced by multiple factors, including environmental, genetic, and behavioral determinants such as alcohol consumption, physical activity, and social relationships [10]. As a pivotal and modifiable factor, diet could also impact sleep health in the elderly [11].

Plant-based foods like vegetables and fruits, which are typically abundant in nutrients like flavonoids and polyphenols, are common in plant-based diets. [12]. Numerous investigations have proved the effect of plant-based diets upon health, reporting that following these patterns is linked to a decreased risk of insomnia, as well as oxidative stress [13]. Evidence from population-based investigation has also identified that plant-based dietary patterns are positively connected to improved cognitive function and diminished cardiovascular disease risk [14]. In addition, a nutritious plant-based diet may increase the availability of tryptophan, an amino acid that subsequently raises melatonin and serotonin metabolite levels, thereby reducing sleep disorders. Hence, we hypothesized that plant-based diets affect sleep quality and duration in the elderly, with differing impacts of the healthy plant-based diet indices (hPDIs) and the uPDI.

As a common mental health issue among the elderly, depression may result in a declined interest in daily life [15,16,17,18]. Another prevalent mental issue among the elderly is anxiety accompanied by an anxious emotional state. Previous research has confirmed a positive association between plant-based diet indices and mental health, with heightened adherence to such dietary patterns being linked to a reduced risk of mental disorders [19]. The potential link between them may be attributed to the rich content of anti-inflammatory compounds in plant-based diets, which could reduce the risk of suffering from depression and anxiety [20]. Furthermore, it can also affect the production of neurotransmitters that are key regulators of mood [21]. Additionally, there is a well-documented inverse relationship between sleep issues and depression or anxiety among the elderly [22]. Older individuals experiencing depression tend to have a poorer quality sleep and experience increased nocturnal awakenings [23]. Concurrently, the elderly with complaints of insomnia may further exacerbate the risk of psychiatric disorders.

Previous research has revealed a connection between plant-based diets and sleep patterns [24]. However, limited research has addressed the potential mediating role of anxiety and depression in this relationship. Therefore, we intended to identify the connection between plant-based diets and sleep health among the elderly, as well as the parallel mediating role of anxiety and depression.

## 2. Materials and Methods

### 2.1. Research Subjects

Data were derived from the CLHLS, which was begun in 1998. This survey, conducted every 3 to 4 years, primarily focuses on participants older than 65 years old. The study employed the multi-stage stratified cluster sampling method, with respondents selected from 22 Chinese provinces [25,26]. The investigation was authorized by the Biomedical Ethics Committee of Peking University, China (IRB00001052-13074). Every respondent submitted informed consent [27]. Detailed descriptions of the CLHLS are available elsewhere [28].

Data were obtained from the 2018 wave of CLHLS. Participants younger than 65 years old, as well as those with missing values for dietary data, anxiety and depression data, sleep quality data, or covariates, were excluded. The final analysis contained 6853 participants (Figure S1).

### 2.2. Outcome Measures

In this study, the dependent variable was sleep health (defined by 2 components: sleep quality and sleep duration), assessed based on 2 questions from the questionnaire: “How is your current sleep quality?” and “How many hours do you generally sleep per day?”. Poor sleep quality was categorized as “fair”, “poor”, or “very poor” and assigned a value of 0, while “very good” and “good” were given a value of 1, indicating good sleep quality. Based upon the update of the National Sleep Foundation’s sleep duration recommendations for older adults, sleep duration was classified into 3 categories: appropriate (7–8 h), short (<7 h), or long (>8 h) [29]. Meanwhile, short or long sleep durations were denoted as 0, while a normal sleep duration was coded as 1.

### 2.3. Measurements of PDI

Plant-based diets were assessed by the PDI [26,30]. Simplified food frequency questionnaires were utilized to gather the dietary information of participants. 16 of the most commonly consumed food groupings were divided into 3 types based upon potential health impacts: animal-based foods (animal fats, meats, fish and seafood, eggs, milk, and dairy products), unhealthy plant-based foods (refined grains, salted vegetables, and sugar), and healthy plant-based foods (whole grains, vegetable oils, fruits, vegetables, legumes, garlic, nuts, and tea) [31,32]. 

Drawing on prior research, the PDI offers a general indicator of plant-based diet intake, while the hPDIs and uPDI allow us to differentiate between healthy and unhealthy plant-based food consumption [33,34]. To evaluate the differential impacts of plant-based diets on sleep health, we incorporated the PDI, hPDIs, and uPDI simultaneously. The 16 food groups were scored on a scale from 1 to 5. For the PDI, plant-based foods received ascending scores from 1 to 5 with increasing frequency, while animal-based foods were inversely scored. The hPDIs assigned positive scores to healthy plant foods, with unhealthy plant and animal foods receiving inverse scores. Conversely, the uPDI gave positive scores to unhealthy plant foods, while healthy plant and animal foods were inversely scored. We calculated the PDI, hPDIs, and uPDI by the summing scores across the 16 food groups. More details about the building of the PDI, hPDIs, and uPDI are accessible in Table S1. The theoretical scores for these indices range from 16 to 80. Higher scores for the PDI and hPDIs indicate better adherence towards plant-based diets, whereas for the uPDI, the opposite is true. In addition, the scores for the PDI, hPDIs, and uPDI were divided into 4 quartiles (Q1, Q2, Q3 and Q4) for analysis. The participant distributions for the hPDI and uPDI quartiles were 1775, 1988, 1535, and 1555, and 2207, 1479, 1784, and 1383, respectively.

### 2.4. Assessment of Depression and Anxiety

Anxiety was assessed utilizing the Generalized Anxiety Disorder (GAD-7) scale, which involves 7 items. A 4-point Likert scale was utilized, where greater scores correlate with elevated levels of anxiety. A score of 5 or above suggests the presence of anxiety. The Cronbach’s alpha coefficient for the GAD-7 was 0.912, demonstrating good internal consistency.

Depression was identified with the Center for Epidemiologic Studies Depression Scale-10 (CES-D-10). The sleep quality items in the CES-D-10 were used to measure sleep health. Thus, the items related to sleep quality were not incorporated, resulting in a shortened version of the CES-D9. Respondents’ responses were classified on a four-point scale: “rarely”, “occasionally”, “frequently”, and “most of the time”. The score for the positive effect item was inverted. The total score of the CES-D-9 ranged from 0 to 27 [35,36,37]. A cutoff score of ≥9 was utilized to identify depression. The Cronbach’s alpha coefficient for the CES-D-9 was 0.785.

### 2.5. Covariates

We included covariates across five domains: demographic characteristics (gender, age, residence), socioeconomic characteristics (living condition, marital status, education, occupation, living resources, economic situation, annual household income), health factors (self-reported health, smoking, drinking, exercise, BMI, hypertension, dyslipidemia, diabetes, heart disease, stroke, and cerebrovascular disease), housing factors (housing nature, housing type, separated bedrooms), and regional factors.

### 2.6. Statistical Analysis

Numbers and percentages were utilized to describe category variables. Mean ± SD was employed to summarize continuous variables. Stata version 18.0 was used to carry out binary logistic regression model to examine the impacts of plant-based diets on sleep health, while controlling for confounding variables. Structural equation modeling (SEM) was performed via AMOS 26.0 to test the parallel mediating effects of anxiety and depression on the connection between plant-based diets and sleep health, with their mediating effects being evaluated using the Bootstrap method [38]. We encompassed both sleep quality and sleep duration as observed variables of sleep health. Additionally, a three-knot restricted cubic spline curve was plotted to explore potential nonlinear relationships between plant-based diets and sleep health [39]. R (version 4.2.3) was employed for RCS analyses, which was carried out utilizing the R package “rms”. And a *p*-value below 0.05 was determined as significance for all tests.

## 3. Results

### 3.1. Sample Characteristics 

Within the 6853 participants, 3585 were women (52.31%), with an average age of 82.46 ± 11.31 years. Urban residents comprised 17.58% of the sample, and nearly 60% had received education. Approximately 17% were current smokers and drinkers. The average BMI was 23.68 kg/m^2^. Anxiety symptoms were present in 10.84% of participants, while 47.32% had depressive symptoms. The baseline traits categorized by PDI groups are exhibited in Table 1.

### 3.2. Association between the PDI and Sleep Health

The regression analysis showed that respondents in the highest PDI quartile had a 1.209-fold increased likelihood of having a better sleep quality (95% CI: 1.039–1.407) as well as a 1.241 times higher likelihood of having an appropriate sleep duration (95% CI: 1.072–1.437) in comparison with the lowest quartile (Table 2). Respondents in the highest uPDI quartile had a 32.2% lower chance of having a good sleep quality (OR: 0.678, 95% CI: 0.574–0.800) and 23.8% decreased chance of having an appropriate sleep duration (OR: 0.762, 95% CI: 0.647–0.896). Similarly, as continuous variables, both the PDI and the hPDI showed positive associations with sleep quality and sleep duration, whereas the uPDI exhibited a significant inverse correlation.

**Figure 1:**
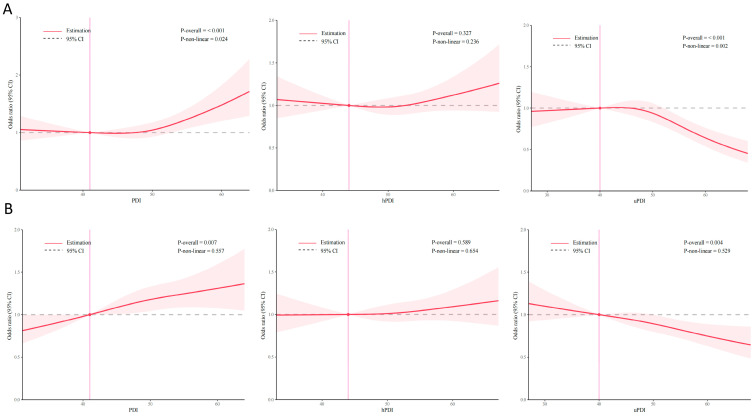
(**A**) The non-linear relationship between the PDI scores and sleep quality. (**B**) The non-linear relationship between the PDI scores and sleep duration.

The results from the RCS indicated that the PDI and the uPDI were significantly related to sleep quality in older adults (*p* for overall < 0.05). The correlations between the PDI, the uPDI, and sleep quality exhibited a nonlinear dose–response relationship (*p* for nonlinear < 0.05). Additionally, a linear dose–response relationship for the PDI and the uPDI with appropriate sleep duration in older adults was also established (*p* for nonlinear > 0.05) (Figure 1).

### 3.3. The Mediated Effect of Anxiety and Depression

**Figure 2:**
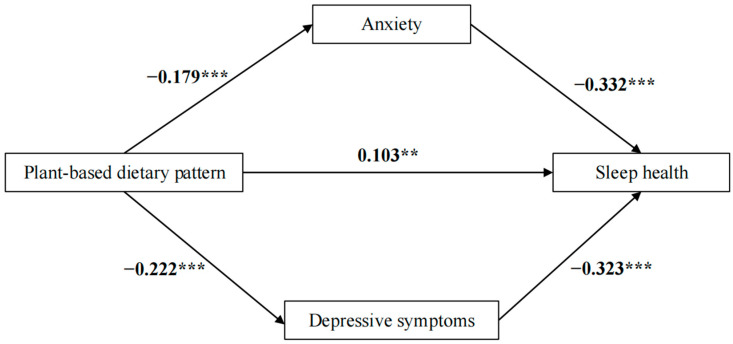
Analysis of the mediation effects. Notes: ** *p* < 0.01; *** *p* < 0.001. The SEM model was adjusted for all covariates.

The mediated roles of anxiety and depression were validated via the bootstrap test (Figure 2). Our findings revealed that depression and anxiety significantly mediated the connection between a plant-based dietary pattern and sleep health. A good model fit was identified via the following results: SRMR = 0.088, TLI = 0.776, RMSEA = 0.051 (95% CI: 0.050–0.051), and CFI = 0.820.

The total impact of a plant-based diet on sleep health was significant (β = 0.234, 95% CI [0.158, 0.307], *p* < 0.01). The direct effect of a plant-based dietary pattern on sleep health was also notable (β = 0.103, 95% CI [0.031, 0.174], *p* < 0.01), representing 44.02% of the total effect. Additionally, the indirect effect of anxiety was notable (β = 0.059, 95% CI [0.043, 0.078], *p* < 0.01), contributing 25.21% to the total effect. Depression had a substantial indirect effect (β = 0.072, 95% CI [0.052, 0.094], *p* < 0.01), representing 30.77% of the total impact (Table S2).

## 4. Discussion

The goal of our investigation was to probe the connection between plant-based diets and sleep health in older adults from China. Additionally, we evaluated the potential mediating roles of anxiety and depression in this connection. The findings illustrated that a higher PDI was connected to improved sleep quality and sleep duration. Moreover, depression and anxiety may serve as parallel mediators between a plant-based dietary pattern and sleep health.

The findings confirmed that a higher adherence to the PDI and the hPDIs was positively correlated with an optimal sleep quality among the elderly. Extant research indicated that sleep health was correlated with maintaining healthful plant-based diets [24]. For example, consuming plant-based foods like soybeans, whole grains, and vegetables may correlate with improved sleep patterns [11,40]. Consistently, a cohort study conducted in elderly and middle-aged adults also identified that higher PDI and hPDIs values were inversely connected to poor sleep efficiency and sleep duration [24]. This connection could be explained by multiple processes. Firstly, a plant-based dietary pattern affluent in vitamins, minerals, antioxidants, and phytochemicals could reduce inflammation and oxidative stress, which was linked to sleep-related issues [41]. Phytochemicals within a plant-based diet, including flavonoids, as well as phytoestrogens like isoflavones and lignans, may also improve sleep patterns. And several plant-based diets like fruits and vegetables are one of the sources of melatonin and tryptophan, which could also enhance sleep health through boosting melatonin and serotonin levels [42].

Consistent with prior research, we found that anxiety and depression directly impair sleep quality among older adults. Psychological health disorders, like depression and anxiety, are among the greatest pressing crises, particularly for the elderly. Evidence has constituted a robust connection between the incident of anxiety and depression and sleep health. A recent investigation discovered that participants diagnosed with anxiety or depression exhibited a higher inclination towards experiencing insomnia and maladaptive sleep behaviors [43]. Evidence also indicated a bidirectional link between depression and sleep quality. The elderly suffering from depression frequently experience a poor sleep quality, while sleep disturbances could further worsen depression [44]. Meanwhile, sleep disturbances are intrinsic to depression, which affected a significant majority of the patients [45]. The bidirectional connection could be clarified by common mechanisms, such as inflammation and hypothalamic–pituitary–adrenal axis dysregulation, which may cause depression and are highly linked to sleep issues [46].

The mediation analysis showed that depression and anxiety mediated the connection between a plant-based dietary pattern and sleep health, which indicates that a plant-based diet affects sleep health both directly and indirectly through depression and anxiety. An investigation from Iran revealed that an unhealthy plant-based diet may be linked to an elevated risk of anxiety or psychological distress [47,48]. The evidence also highlighted the preventive impacts of plant-based diets towards psychological disorders [49]. Individuals with a higher hPDI, characterized by consuming a lot of whole grains, fruits, vegetables, olive oil, or antioxidants, showed lower depression risk [50]. Furthermore, dietary polyphenols found in plant-based diets, like lignans and phenolic acids, were proposed to offer protection from depression [51]. Furthermore, poor sleep effectiveness and satisfaction were also strongly linked to a depressed mood [52]. Thus, a plant-based diet may improve mental health in the elderly, which in turn reduces the incidence of mood disorders and indirectly alleviates sleep disorders, leading to better sleep health.

This study possesses multiple advantages. Primarily, this study identified depression and anxiety as parallel mediators between plant-based diets and sleep health, offering potential targeted interventions to improve sleep health among the elderly. Additionally, by adjusting the wide range covariates and incorporating major potential confounders, this study provided a profound comprehension of the connection between plant-based diets and sleep health.

This research also had certain shortcomings. First, the cross-sectional design of this data prevents causality inferences toward the connection between plant-based diets and sleep health, as well as the mediating effects identified. Second, recall bias could be generated via self-reported data. Additionally, due to the constraints of the CLHLS database, our research utilized only two items related to sleep quality and sleep duration [35,37]. A single item for sleep duration may not capture the full complexity of sleep patterns, including the distinction between 24 h and nocturnal sleep. Future studies should incorporate more sleep scales to enhance the accuracy of assessment. Furthermore, while the CES-D9 scale achieved satisfactory psychometric properties, the cutoff score of ≥9 may result in an overestimation of depression detection rates. Future studies could employ multiple scales to further identify the prevalence in older adults. Specifically, the relationship between mental health and sleep is bidirectional. We prioritized the investigation of effects of mental health on sleep rather than the reverse, which restricted providing additional insights into the complex interactions. Future designs should also consider the potential for sleep health to influence mental health.

## 5. Conclusions

In summary, a plant-based dietary pattern promotes better sleep quality and sleep duration in Chinese elderly adults, with depression and anxiety partially mediating this relationship. Given the rapid aging of the Chinese population, these findings can inform targeted preventive measures. Public policies could prioritize the psychological health as well as dietary habits of older adults to enhance their sleep health.

## Figures and Tables

**Table 1:** Characteristics of respondents across PDI groups.

Characteristics	Total (n = 6853)	Q1 (n = 1938)	Q2 (n = 1460)	Q3 (n = 1887)	Q4 (n = 1568)	*p* Value
Anxiety	743 (10.84)	260 (34.99)	156 (21.00)	193 (25.98)	134 (18.03)	0.000
Depression	3243 (47.32)	1053 (32.47)	720 (22.20)	881 (27.17)	589 (18.16)	0.000
Gender						
Female	3585 (52.31)	1102 (30.74)	776 (21.65)	984 (27.45)	723 (20.17)	0.000
Male	3268 (47.69)	836 (25.58)	684 (20.93)	903 (27.63)	845 (25.86)	
Age *	82.46 (11.31)	85.15 (11.31)	82.99 (11.30)	81.35 (11.03)	80.00 (10.89)	0.000
Residence						
Rural	5648 (82.42)	1656 (29.32)	1190 (21.07)	1542 (27.30)	1260 (22.31)	0.000
Urban	1205 (17.58)	282 (23.40)	270 (22.41)	345 (28.63)	308 (25.56)	
Living condition						
Live with family	5719 (83.45)	1559 (27.26)	1227 (21.45)	1593 (27.85)	1340 (23.43)	0.001
Solitude	1123 (16.39)	375 (33.39)	229 (20.39)	294 (26.18)	225 (20.04)	
Other	11 (0.16)	4 (36.36)	4 (36.36)	0 (0.00)	3 (27.27)	
Marital status						
Married/cohabitating	3326 (48.53)	776 (23.33)	697 (20.96)	975 (29.31)	878 (26.40)	0.000
Widowed	3365 (49.10)	1115 (33.14)	734 (21.81)	863 (25.65)	653 (19.41)	
Other	162 (2.36)	47 (29.01)	29 (17.90)	49 (30.25)	37 (22.84)	
With formal education	4048 (59.07)	959 (23.48)	887 (21.91)	1150 (28.41)	1052 (25.99)	0.000
Occupation						
Professional and technical personnel	542 (7.90)	93 (17.16)	124 (22.88)	157 (28.97)	168 (31.00)	0.000
Governmental, institutional, or managerial personnel	317 (4.63)	63 (19.87)	68 (21.45)	88 (27.76)	98 (30.91)	
General staff, service personnel or workers	1081 (15.77)	253 (23.40)	247 (22.85)	311 (28.77)	270 (24.98)	
Farmers	4129 (60.25)	1310 (31.73)	854 (20.68)	1127 (27.29)	838 (20.30)	
Other	784 (11.44)	219 (27.93)	167 (21.30)	204 (26.02)	194 (24.74)	
Sufficiency of living resources	6036 (88.08)	1626 (26.94)	1305 (21.63)	1693 (28.05)	1412 (23.39)	0.000
Wealthy economic situation	1457 (21.26)	324 (22.24)	318 (21.83)	418 (26.69)	397 (27.25)	0.000
Annual household income *	43,507.55 (36,920.75)	39,313.31 (35,421.25)	44,861.19 (37,496.54)	44,753.63 (37,183.04)	45,931.50 (37,487.84)	0.000
Housing nature						
Purchased or self-built	5985 (87.33)	1701 (28.42)	1269 (21.21)	1652 (27.60)	1363 (22.77)	0.831
Other	868 (12.67)	237 (27.30)	191 (22.00)	235 (27.07)	205 (23.62)	
Housing type						
Bungalow	637 (9.30)	241 (37.83)	114 (17.90)	183 (28.73)	99 (15.54)	0.000
Apartment	2009 (29.32)	516 (25.68)	423 (21.06)	545 (27.13)	525 (26.13)	
Other	4207 (61.39)	1181 (28.07)	923 (21.94)	1159 (27.55)	944 (22.44)	
Separated bedrooms	6503 (94.89)	1830 (28.14)	1376 (21.16)	1808 (27.80)	1489 (22.90)	0.142
Self-reported health						
Poor	873 (12.74)	310 (35.51)	195 (22.34)	206 (23.60)	162 (18.56)	0.000
Fair	2567 (37.46)	794 (30.93)	595 (23.18)	679 (26.45)	499 (19.44)	
Good	3413 (49.80)	834 (24.44)	670 (19.63)	1002 (29.36)	907 (26.57)	
Smoking						
Never	4631 (67.58)	1396 (30.14)	1006 (21.72)	1301 (28.09)	928 (20.04)	0.000
Former	1078 (15.73)	247 (22.91)	228 (21.15)	280 (25.97)	323 (29.96)	
Current	1144 (16.69)	295 (25.79)	226 (19.76)	306 (26.75)	317 (27.71)	
Drinking						
Never	4895 (71.43)	1424 (29.09)	1081 (22.08)	1367 (27.93)	1023 (20.90)	0.000
Former	820 (12.01)	242 (29.51)	156 (19.02)	218 (26.59)	204 (24.88)	
Current	1138 (16.61)	272 (23.90)	223 (20.47)	302 (26.54)	341 (29.96)	
Exercise						
Never	3870 (56.47)	1232 (31.83)	868 (22.43)	1043 (26.95)	727 (18.76)	0.000
Former	487 (7.11)	152 (31.21)	101 (20.74)	120 (24.64)	114 (23.41)	
Current	2496 (36.42)	554 (22.20)	491 (19.67)	724 (29.01)	727 (29.13)	
BMI (kg/m^2^) *	23.68 (36.72)	23.15 (20.52)	23.14 (14.87)	24.93 (65.30)	23.34 (5.85)	0.000
Hypertension	2986 (43.57)	745 (24.95)	648 (21.70)	874 (29.27)	719 (24.08)	0.000
Diabetes	560 (8.17)	131 (23.39)	147 (26.25)	163 (25.08)	119 (21.25)	0.004
Heart disease	1231 (17.96)	315 (25.59)	271 (22.01)	361 (29.33)	284 (23.07)	0.113
Stroke, cerebrovascular disease	748 (10.91)	180 (24.06)	172 (22.99)	230 (30.75)	166 (22.19)	0.021
Dyslipidemia	468 (6.83)	93 (19.87)	114 (24.36)	129 (27.56)	132 (28.21)	0.000
Geographic region						
East China	3317 (48.40)	747 (22.52)	713 (21.50)	989 (29.82)	868 (26.17)	0.000
Central China	1568 (22.88)	466 (29.72)	366 (23.34)	432 (27.55)	304 (19.39)	
West China	1608 (23.46)	649 (40.36)	314 (19.53)	348 (21.64)	297 (18.47)	
Northeast China	360 (5.25)	76 (21.11)	67 (18.61)	118 (32.78)	99 (27.50)	

Notes: Numbers (%) were reported; * mean (standard deviation) was reported.

**Table 2:** Connections between a plant-based dietary pattern and sleep health.

	Continuous	Q1	Q2	Q3	Q4	*p* for Trend
	OR (95% CI)		OR (95% CI)	OR (95% CI)	OR (95% CI)	
Sleep quality						
PDI						
Model 1	**1.037 (1.028, 1.046)**	1.0 (ref.)	1.104 (0.964, 1.264)	**1.269 (1.118, 1.442)**	**1.676 (1.464, 1.919)**	**<0.001**
Model 2	**1.014 (1.003, 1.024)**	1.0 (ref.)	0.972 (0.837, 1.128)	1.029 (0.894, 1.184)	**1.209 (1.039, 1.407)**	**0.017**
hPDI						
Model 1	**1.025 (1.016, 1.035)**	1.0 (ref.)	**1.171 (1.030, 1.331)**	**1.262 (1.101, 1.448)**	**1.420 (1.238, 1.630)**	**<0.001**
Model 2	1.004 (0.993, 1.014)	1.0 (ref.)	1.013 (0.880, 1.166)	1.002 (0.860, 1.167)	1.076 (0.921, 1.256)	0.413
uPDI						
Model 1	**0.962 (0.956, 0.969)**	1.0 (ref.)	0.911 (0.797, 1.041)	**0.744 (0.656, 0.844)**	**0.474 (0.413, 0.543)**	**<0.001**
Model 2	**0.982 (0.973, 0.990)**	1.0 (ref.)	1.023 (0.880, 1.188)	0.926 (0.797, 1.075)	**0.678 (0.574, 0.800)**	**<0.001**
Sleep duration						
PDI						
Model 1	**1.033 (1.024, 1.043)**	1.0 (ref.)	**1.199 (1.040, 1.382)**	**1.405 (1.231, 1.603)**	**1.548 (1.349, 1.776)**	**<0.001**
Model 2	**1.017 (1.007, 1.027)**	1.0 (ref.)	1.086 (0.937, 1.258)	**1.204 (1.049, 1.382)**	**1.241 (1.072, 1.437)**	**0.001**
hPDI						
Model 1	**1.026 (1.017, 1.036)**	1.0 (ref.)	1.073 (0.938, 1.227)	**1.351 (1.173, 1.556)**	**1.412 (1.227, 1.625)**	**<0.001**
Model 2	1.007 (0.997, 1.017)	1.0 (ref.)	0.942 (0.820, 1.083)	1.107 (0.955, 1.283)	1.100 (0.947, 1.278)	0.057
uPDI						
Model 1	**0.969 (0.962, 0.976)**	1.0 (ref.)	**0.765 (0.669, 0.875)**	**0.680 (0.598, 0.773)**	**0.564 (0.490, 0.650)**	**<0.001**
Model 2	**0.985 (0.977, 0.994)**	1.0 (ref.)	0.869 (0.754, 1.002)	**0.819 (0.709, 0.946)**	**0.762 (0.647, 0.896)**	**0.001**

Notes: Q, quartile. Model 1 was a crude model. Model 2 was adjusted for gender, age, residence, living condition, marital status, education, occupation, living resources, economic situation, annual household income, housing nature, housing type, separated bedrooms, self-reported health, smoking, drinking, exercise, BMI, hypertension, diabetes, heart disease, stroke, cerebrovascular disease, dyslipidemia, and region. The bold values represent significant odds ratios (ORs), corresponding to *p*-values less than 0.05.

## Data Availability

The data that support the findings of this study are openly available in Chinese Longitudinal Healthy Longevity Survey (CLHLS) at https://opendata.pku.edu.cn/dataverse/CHADS (accessed on 16 January 2024). Further inquiries can be directed to the corresponding authors.

## Supplementary Materials

The following supporting information can be downloaded at: https://www.mdpi.com/article/10.3390/nu16193386/s1, Table S1. Plant-based diet index scoring; Table S2. The results of parallel mediation effect model; Figure S1. Flowchart of the study population.
