# Supplementary material for: The Relationship between Plant-Based Diet Indices and Sleep Health in Older Adults: The Mediating Role of Depressive Symptoms and Anxiety
Source: Nutrients. 2024 Oct 5;16(19):3386. doi: 10.3390/nu16193386 (PMC11478969; doi:10.3390/nu16193386)
Supplement: Supplementary file 1 [file nutrients-16-03386-s001.zip › nutrients-3188638-supplementary.pdf]

Supplementary Materials

Table S1. Plant-based diet index scoring

Table S2. The results of parallel mediation effect model.

Figure S1. Flowchart of the study population.

Table S1. Plant-based diet index scoring

Food	Food	Frequency	PDI score	hPDI score	uPDI score
Plant food	Whole grain	Yes	5	5	1
		No	1	1	5
	Vegetable oil	Yes	5	5	1
		No	1	1	5
	Fresh fruit	Almost everyday	5	5	1
		Quite often	4	4	2
		Occasionally	2	2	4
		Rarely or never	1	1	5
	Fresh vegetable	Almost everyday	5	5	1
		Quite often	4	4	2
		Occasionally	2	2	4
		Rarely or never	1	1	5
	Legume	Almost everyday	5	5	1
		≥1 time/week	4	4	2
		≥1 time/month	3	3	3
		Occasionally	2	2	4
		Rarely or never	1	1	5
	Garlic	Almost everyday	5	5	1
		≥1 time/week	4	4	2
		≥1 time/month	3	3	3
		Occasionally	2	2	4
		Rarely or never	1	1	5
	Nut	Almost everyday	5	5	1
		≥1 time/week	4	4	2
		≥1 time/month	3	3	3
		Occasionally	2	2	4
		Rarely or never	1	1	5
	Tea	Almost everyday	5	5	1
		≥1 time/week	4	4	2
		≥1 time/month	3	3	3
		Occasionally	2	2	4
		Rarely or never	1	1	5
	Refined grain	Yes	5	1	5
		No	1	5	1
	Sugar	Almost everyday	5	1	5
		≥1 time/week	4	2	4
		≥1 time/month	3	3	3
		Occasionally	2	4	2
		Rarely or never	1	5	1
	Salt-preserved vegetable	Almost everyday	5	1	5
		≥1 time/week	4	2	4
		≥1 time/month	3	3	3
		Occasionally	2	4	2
		Rarely or never	1	5	1
		Yes	1	1	1

Animal food	Animal fat	No	5	5	5
	Meat	Almost everyday	1	1	1
		≥1 time/week	2	2	2
		≥1 time/month	3	3	3
		Occasionally	4	4	4
		Rarely or never	5	5	5
	Fish	Almost everyday	1	1	1
		≥1 time/week	2	2	2
		≥1 time/month	3	3	3
		Occasionally	4	4	4
		Rarely or never	5	5	5
	Egg	Almost everyday	1	1	1
		≥1 time/month	3	3	3
		Occasionally	4	4	4
		Rarely or never	5	5	5
	Dairy products	Almost everyday	1	1	1
		≥1 time/week	2	2	2
		≥1 time/month	3	3	3
		Occasionally	4	4	4
		Rarely or never	5	5	5

Abbreviations: PDI: plant-based diet index; hPDI: healthful plant-based diet index; uPDI: unhealthful plant-based diet index.

Table S2. The results of parallel mediation effect model.

Model pathways	Estimate	S.E.	95% CI		*P* value
			Lower	Upper	
Total effect	0.234	0.038	0.158	0.307	0.000
Direct effect	0.103	0.036	0.031	0.174	0.005
Specific indirect effects					
Indirect effect 1	0.059	0.009	0.043	0.078	0.000
Indirect effect 2	0.072	0.011	0.052	0.094	0.000
Total indirect effects	0.131	0.014	0.104	0.161	0.000

Abbreviations: CI, confidence intervals.

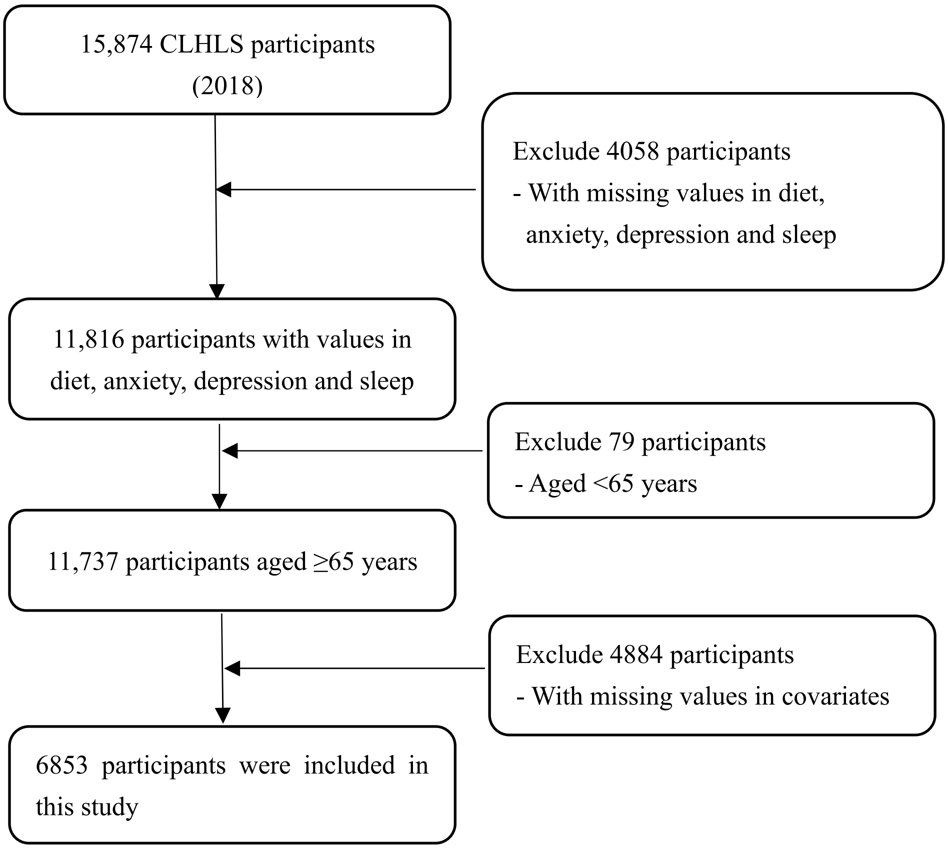

Figure S1. Flowchart of the study population.
